# Real-Time *In Vivo* Imaging of Butterfly Wing Development: Revealing the Cellular Dynamics of the Pupal Wing Tissue

**DOI:** 10.1371/journal.pone.0089500

**Published:** 2014-02-21

**Authors:** Masaki Iwata, Yoshikazu Ohno, Joji M. Otaki

**Affiliations:** The BCPH Unit of Molecular Physiology, Department of Chemistry, Biology and Marine Science, Faculty of Science, University of the Ryukyus, Nishihara, Okinawa, Japan; University of Cincinnati, United States of America

## Abstract

Butterfly wings are covered with regularly arranged single-colored scales that are formed at the pupal stage. Understanding pupal wing development is therefore crucial to understand wing color pattern formation. Here, we successfully employed real-time *in vivo* imaging techniques to observe pupal hindwing development over time in the blue pansy butterfly, *Junonia orithya*. A transparent sheet of epithelial cells that were not yet regularly arranged was observed immediately after pupation. Bright-field imaging and autofluorescent imaging revealed free-moving hemocytes and tracheal branches of a crinoid-like structure underneath the epithelium. The wing tissue gradually became gray-white, epithelial cells were arranged regularly, and hemocytes disappeared, except in the bordering lacuna, after which scales grew. The dynamics of the epithelial cells and scale growth were also confirmed by fluorescent imaging. Fluorescent *in vivo* staining further revealed that these cells harbored many mitochondria at the surface of the epithelium. Organizing centers for the border symmetry system were apparent immediately after pupation, exhibiting a relatively dark optical character following treatment with fluorescent dyes, as well as in autofluorescent images. The wing tissue exhibited slow and low-frequency contraction pulses with a cycle of approximately 10 to 20 minutes, mainly occurring at 2 to 3 days postpupation. The pulses gradually became slower and weaker and eventually stopped. The wing tissue area became larger after contraction, which also coincided with an increase in the autofluorescence intensity that might have been caused by scale growth. Examination of the pattern of color development revealed that the black pigment was first deposited in patches in the central areas of an eyespot black ring and a parafocal element. These results of live *in vivo* imaging that covered wide wing area for a long time can serve as a foundation for studying the cellular dynamics of living wing tissues in butterflies.

## Introduction

The spectacular diversity of butterfly wing color patterns has fascinated many evolutionary biologists throughout the history of biological research, but it was only after the seminal work of Nijhout [1] that biologists began to discuss evolutionary developmental aspects of butterfly wing color pattern formation. Despite their diversity, the color patterns of nymphalid butterfly wings appear to be constructed according to the nymphalid groundplan [1]–[3], which is a general rule underlying color pattern development and evolution. The nymphalid groundplan is the scheme in which color pattern elements are placed in a plain background. The groundplan is basically composed of three major symmetry systems (the basal, central, and border symmetry systems) and two peripheral systems (the wing root band system and the marginal band system) [3]. At least the major symmetry systems are composed of a core element and a pair of paracore elements that surround the core [3]. In addition to these color pattern elements, there are venous stripes, intervenous striples, ripple patterns, and background coloration, which are not understood within the scheme of the nymphalid groundplan [1].

These various color patterns are composed of the scales that cover the surface of butterfly wings. Each scale is produced by a single scale cell and exhibits a single distinct color [1], [4], [5], which may be dubbed the “one cell, one scale, and one color” rule. Furthermore, scales are arranged regularly in anteroposterior rows, in parallel to one another and to the outer wing margin [1], [5]–[8]. Two types of scales usually alternate in a row: cover and ground scales [1], [5]–[8]. The arrangement of rows occurs at the pupal stage, and it does not appear to contribute to the determination of color pattern elements [5]–[8].

Among the color pattern elements that constitute the overall wing color pattern, the most conspicuous are likely eyespots, which belong to the border symmetry system. A species of butterfly that has been used to study eyespot formation, especially in early research, is the American buckeye butterfly, *Junonia coenia* [9]–[11], which is distributed in North America. Since the 1990s, many studies on the development of butterfly wing color patterns have focused on the eyespot structure of the African satyrine butterfly, *Bicyclus anynana* [12]–[14]. Additionally, we have been using several species of *Junonia* and other butterflies to study the mechanistic basis of color pattern diversity, such as the blue pansy, *J. orithya* [15]–[17], and the peacock pansy, *J. almana* [18]–[20], both of which are distributed widely in Southeast Asia, including on the Ryukyu Archipelago of Japan. These species display large eyespots on their wings, which facilitate color pattern analysis.

Surgical studies have demonstrated that the prospective eyespot focus functions as an organizing center for an eyespot [1], [10]–[13], [19], [21]. It is likely that the same organizing center is responsible for the entire border symmetry system, including an eyespot (i.e., the core element) and parafocal elements (i.e., paracore elements), despite their partial independence [3], [21]–[24]. Because of the developmental function of the prospective eyespot focus as an organizing center, many studies in butterflies have focused on putative molecular mechanisms underlying eyespot development. These studies have identified several candidate genes, such as *Distal-less*, that may be responsible for color pattern formation [25]–[31]. Although sequence variation in *Distal-less* is reported to be correlated with size variation in eyespots [27], the functional status of *Distal-less* is not yet fully understood due to a lack of sufficient molecular evidence of its organizing activities despite a considerable effort to produce transgenic butterflies [31].

In contrast to these molecular approaches, we have been studying morphological and physiological aspects of butterfly wing development and color pattern determination [32]–[34]. We have proposed that in addition to the organizing centers for eyespots (i.e., the border symmetry system), organizing centers for other symmetry systems also exist on the surface of a developing wing [21]. Interestingly, it appears that these organizing centers can be identified based on the presence of pupal cuticle spots in many nymphalid butterflies [21]. For example, one of the groups of pupal cuticle spots, referred to as edge spots, are found along the outer margin [3], [21]. The edge-spot cells may function as organizing centers for the marginal band system [3], [21].

To understand the physiological aspects of color pattern development at the cellular and tissue levels, basic descriptive records of normally developing pupal wings are necessary as a foundation for interpreting any experimental data. We know how the development of butterfly wings occurs in the larval and pupal stages based on morphological and histochemical studies [1], [35], [36]. First, in larvae, epidermal cells form the wing imaginal discs, which then form a sac-like structure consisting of a single cellular sheet [35]. This sac-like structure is flattened but leaves gaps as lacunae. Tracheae then elongate into lacunae. At the time of pupation, the larval wings (i.e., the wing imaginal discs) expand extensively, and the pupal wings already display the primary tracheal system that later merges with the wing veins. At this point, although the sheet of epithelial cells consists of a monolayer, they are not arranged regularly. These epithelial cells undergo cell division, forming one daughter precursor that gives rise to scale and socket cells and another daughter cell that undergoes programmed cell death [36], [37]. The precursor cells then undergo another cell division, horizontally in this case, producing scale and socket cells [36], [37]. At this point, the scale and socket cells are arranged regularly in rows [6]–[8]. Scale cells are likely organized via Notch-mediated lateral inhibition process, similarly to bristles in other insects [38], [39]. The wing boundary is determined by the bordering lacuna, and the peripheral tissue outside the bordering lacuna degenerates [40]–[44].

While we can reconstruct these developmental sequences, the previous studies on lepidopteran wings were performed using fixed tissues at given time points. Partly to compensate for this approach, we have previously analyzed the scale number, size, shape, and arrangement in adult wings to infer developmental changes that might have occurred at the pupal stage [45], [46]. Although these morphometric studies are important, it is desirable to directly record the dynamic cellular changes occurring in the rapidly developing wing tissues *in vivo*. For this purpose, we previously developed a simple surgical (but non-invasive) method to expose a developing hindwing inside a pupal case to allow real-time *in vivo* observations to be made [45]. We reported the developmental sequences of single individuals by presenting images obtained at approximately one-hour intervals [45].

The present study is a systems biology approach to the butterfly wing system, which aims at the comprehensive description of components and dynamics of the system. Here, to understand the pupal wings as a biological system, we employed state-of-the-art digital imaging technologies to record real-time *in vivo* images of wing tissue development over time in pupae using *J. orithya*. We employed three different imaging systems, depending on the magnification and optical conditions: a digital single-lens reflex camera system to obtain macroscopic whole-wing images, a high-resolution high-depth digital microscope system to record bright-field images at the compartmental level, and a real-time confocal microscope system for fluorescent imaging at the compartmental and cellular levels. These methods allowed us to observe various characteristics of developing pupal wings in real-time *in vivo* images. In addition to confirming previously known phenomena, we discovered dynamic movements of the wing system that could not be inferred from fixed specimens. Our results build a foundation for in-depth analyses of the developing wing systems in butterflies.

## Materials and Methods

### Ethics Statement

No specific permissions were required to collect the blue pansy (peacock pansy) butterfly, *J. orithya*, in the locations where this study was conducted. This species is one of the most common butterflies in Okinawa, and it is not an endangered or protected species.

### Butterflies

The blue pansy butterfly, *J. orithya* (Lepidoptera, Nymphalidae, Nymphalinae), was used throughout this study. Adult females were caught on Okinawa-jima Island and Ishigaki-jima Island, from which eggs were collected. Additionally, larvae were collected from fields. Larvae were reared at ambient temperatures, approximately 27°C, using a natural host plant, *Plantago major* or *P. asiatica*.

### Operation for Hindwing Exposure

The surgical method that exposes the surfaces of the dorsal hindwing and the ventral forewing for real-time observations was performed as follows. Immediately after pupation (within 1 h after pupation), a pupae was laid down with wing side up, and the tip of a right or left pupal forewing was picked up using forceps. The right or left pupal forewing was then lifted up slowly from the wing tip to the wing base. It is important not to lift up both the forewing and hindwing together and not to physically damage the wings. The exposed hindwing and forewing surfaces were immediately covered with a sheet of transparent plastic wrap or with a piece of cover glass to avoid evaporation of fluid. The operated pupae were allowed to develop into adults at ambient temperatures (approximately 27°C). The entire operation took less than 10 minutes, which made it possible to record images of pupae at 0 h postpupation. Most of the operated pupae developed normal color patterns in the pupal case and then eclosed, although their wings could not expand normally.

### Whole-wing Bright-field Imaging

For whole-wing bright-field macroscopic imaging, a Canon EOS 40D digital single-lens reflex camera with a Canon EF-S 60 mm macrolens was utilized, which was controlled with Canon ZoomBrowser EX and EOS Utility software (Tokyo, Japan), installed in a Fujitsu FM V-Biblo NF/A50 personal computer with the Windows Vista operating system. Automatic shuttering was set to obtain images at 1-min intervals at ISO800, a shutter speed of 1/5, and a diaphragm setting of F10. The focus was adjusted manually.

Static images were compiled as a movie. First, the images were converted from jpeg (1,936×1,288 pixels) to bmp (720×480 pixels) files using the free software JTrim. The converted bmp images were compiled as an avi movie using the free software AviUtl. The avi movie was then compressed and converted into an mp4 using Windows Movie Maker. One of the movies made in this manner is shown as Movie S1 in the present study. The frame rate (frames per second; fps) of this movie is 29.97 fps. Because images were captured at 1-min intervals, 1 sec of the playing movie corresponds to 0.5 h of the real time span.

### Compartmental Bright-field Imaging

High-resolution high-depth wing images at the compartmental level were obtained using a high-resolution high-depth Keyence VHX-1000 and VHX-2000 digital microscopes (Osaka, Japan). Following the operation for hindwing exposure, a pupa was physically fixed in a Petri dish, using double-sided adhesive tape if necessary. To avoid optical reflection from the wing surface, a polarized illumination adaptor was employed, and the dark-field command built into the machine was used. Images were obtained in 2-min or 5-min intervals, focusing on the compartments CuA_1_ (150×) and Sc+R_1_ (400×).

Static images were compiled as a movie. First, the images were converted from jpeg (1,600×1,200 pixels) to bmp (720×480 pixels) files using the free software JTrim or IrfanView. The converted bmp images were then compiled as an avi movie using the free software AviUtl and then converted into an mp4 using Windows Movie Maker. Three of the movies made in this manner are shown as Movies S2, S4, and S6 in the present study. The frame rate of these movies is 29.97 fps. Because the images in Movies S2 and S4 were captured every 5 or 2 min, respectively, 1 sec of the playing movie corresponds to 2.5 or 1 h of the real time span, respectively. Movie S6 was made in the same way as Movie S4, except that the images were obtained from different compartments (Movie S4 from Sc+R_1_ and Movie S6 from CuA_1_).

### Compartmental Autofluorescent Images

Higher magnification compartmental and cellular images were collected using a real-time confocal microscope imaging system including a Nikon Eclipse Ti-U inverted epifluorescence microscope (Tokyo, Japan), a Yokogawa laser-scanning unit CSU-X1 (Tokyo, Japan), a Hamamatsu Photonics ImagEM C9100-13 electron-multiplying charge-couple device (EM-CCD) camera (Hamamatsu, Japan), and the Hamamatsu Photonics AQUACOSMOS/RATIO system (Hamamatsu, Japan). The hindwing was exposed within 0.5 h postpupation. The exposed hindwing was placed on a piece of cover glass (0.12–0.17 mm in thickness; Muto Pure Chemicals, Tokyo, Japan) and subjected to real-time time-lapse confocal imaging. The exposure time was set to 10 sec (14 mW, EM gain 255) with autofluorescent signals being recorded at 1-min intervals using a 488 nm excitation light and a 520/25 nm bandpass filter.

Static images were acquired using AQUACOSMOS 2.6 as naf images (512×512 pixel), which were converted to avi files with the same software. These images were then converted and compressed into mp4 files using the free software Freemake Video Converter. One of the movies made in this way is shown as Movie S3 in the present study. Images were captured at 1-min intervals, but only images collected every 20 min were incorporated into the movie. The frame rate of this movie was set to 12 fps, and 1 sec of this movie therefore corresponds to 240 min (4 h).

### Compartmental and Cellular Fluorescent Images

CFSE (5- or 6-(*N*-Succinimidyloxycarbonyl)-fluorescein 3′, 6′-diacetate) was purchased from Dojindo Molecular Technologies (Kumamoto, Japan). CFSE is useful for tracing cellular dynamics over a relatively long period of time because it remains inside the cell for days. CFSE was dissolved in DMSO (dimethyl sulfoxide) and then in Insect Ringer’s solution (NaCl 10.93 g, KCl 1.57 g, CaCl_2_·2H_2_O 0.83 g, and MgCl_2_·6H_2_O 0.83 g per liter) to obtain a final concentration of 10 µM. The pupal operation was performed similarly as described above, but to load CFSE (and the other fluorescent dyes indicated below), the loading solution (40 µL) was sandwiched between the fore- and hindwings in a similar manner to the previous method for loading various chemicals [22]. The sandwiched state was maintained in a humidified chamber for 30 min to allow loading at ambient temperatures (approximately 27°C). After this step, the loading solution was washed off with Insect Ringer’s solution. The forewing was then curled up again, and the hindwing was placed on a cover glass (0.12–0.17 mm in thickness; Muto Pure Chemicals).

Real-time *in vivo* confocal images of wing compartments following CFSE loading were obtained using the imaging system described above, with a 488 nm excitation light and a bandpass filter of 520/25 nm. We first obtained *Z*-axis scanning images at 5.0-µm sectioning steps (11 images per sampling time point) using a Physik Instrumente P721 PIFCO Piezo Flexure Objective Scanner (Karlsruhe, Germany), which controlled the *Z*-axis position of the objective lens (Nikon CFI S Fluor 10×, [NA] = 0.5). The exposure time for capturing each optical slice was set at 300 msec (6 mW, EM gain 180). One well-focused image per time point was selected out of 11 images. These images were collected at 10-min intervals. The same image depth (*Z*-axis position) and fluorescent intensity were employed at 50 time points (500 min). That is, the depth and intensity were adjusted in every group of 50 time points, so that well-focused images with a reasonable fluorescence intensity could be obtained. These selected images were connected and converted into a movie as described above. One of the movies made in this way is shown as Movie S5 of the present work. The frame rate of this movie was 30 fps. Because images were taken every 10 min, 1 sec of this movie corresponds to 300 min (5 h).

Other fluorescent images were acquired similarly. The final concentrations of the fluorescent dyes were as follows: 100 µM MitoTracker Orange CMTMRos (Life Technologies, Carlsbad, CA, USA), 100 µM SYBR Green-1 (Life Technologies), and 10 µM DiBAC_4_(3) (Bis(1,3-dibutylbarbituric acid)trimethine oxonol, sodium salt) (Dojindo Molecular Technologies). MitoTracker Orange and SYBR Green-1 stain mitochondria and nuclei, respectively. DiBAC_4_(3) is generally used as a membrane potential-sensitive dye. To load DiBAC_4_(3), the curled wing tissue was floated in the solution in a 35-mm glass-base Petri dish with a bottom consisting of a glass plate with a thickness of 0.16–0.19 mm and a diameter of 12 mm (Iwaki, AGC Techno Glass, Tokyo, Japan). Live images of the floating wings were obtained. The excitation and emission wavelengths used to obtain these confocal images were 561 nm and 617/73 nm for MitoTracker Orange and 488 nm and 520/25 nm for SYBR Green-1 and DiBAC_4_(3), respectively.

### Area and Distance Measurements

The relative areas of the compartments and the relative distances between wing veins were measured from acquired images using the free software ImageJ. For these measurements, one movie consisting of whole-wing bright-field images was used to obtain area values (Movie S1), while one consisting of autofluorescent images (Movie S3) and one consisting of compartmental bright-field images (Movie S6) were used to acquire distance values. Reference values were set to indicate the relative values of compartmental changes over time. During these measurements, image distortions were ignored, which were unavoidable due to the expansion of the wing tissue.

## Results

### Whole-wing Development Over Time

**Figure 1:**
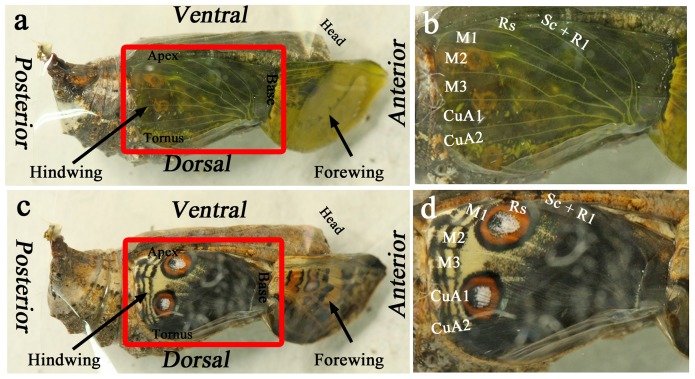
Surgical configuration for observing developing pupal wing tissues *in vivo*. For the complete developmental process over time, refer to Movie S1. (**a**) A pupa immediately after the operation of curling up the pupal forewing. The exposed hindwing is boxed. (**b**) The exposed hindwing from (a), with identification of the compartments. (**c**) A pupa immediately before eclosion with fully developed color patterns on the hindwing. Note that the ventral forewing also display a fully developed color pattern in this individual. This individual is identical to that shown in (a). (**d**) The hindwing of (c), with identification of the compartments, which can be compared to (b). Note that compartment CuA_1_ exhibits a large eyespot.

We first recorded macroscopic live wing images in a developing pupa from the early pupal stage to eclosion using a time-lapse image-capturing method (*n* = 3) (Figure 1; Movie S1). As reported previously [45], the wing tissue was transparent, and the major wing-vein-associated tracheae had already been established at 0 h postpupation (Figure 1a, b). The wing tissue then gradually became gray-white (Movie S1), likely suggesting scale growth. We were able to observe the completed adult wings inside the pupal case prior to eclosion (Figure 1c, d; Movie S1), demonstrating that the normal developmental processes took place throughout the imaging period following the wing-curling operation [45]. We will discuss the developmental changes in color pigment deposition in the scales later in this paper.

We discovered that the wing tissue exhibited contraction pulses from 20 to 80 h postpupation, with a contraction cycle of approximately 10–20 min (Movie S1). The forewing and hindwing contracted synchronously (Movie S1). These slow contraction pulses gradually became weak and finally ceased, and the tissue became almost completely gray-white. These contraction pulses will be examined later in this paper.

### Dynamics at the Compartmental Level over the First Two Days

**Figure 2:**
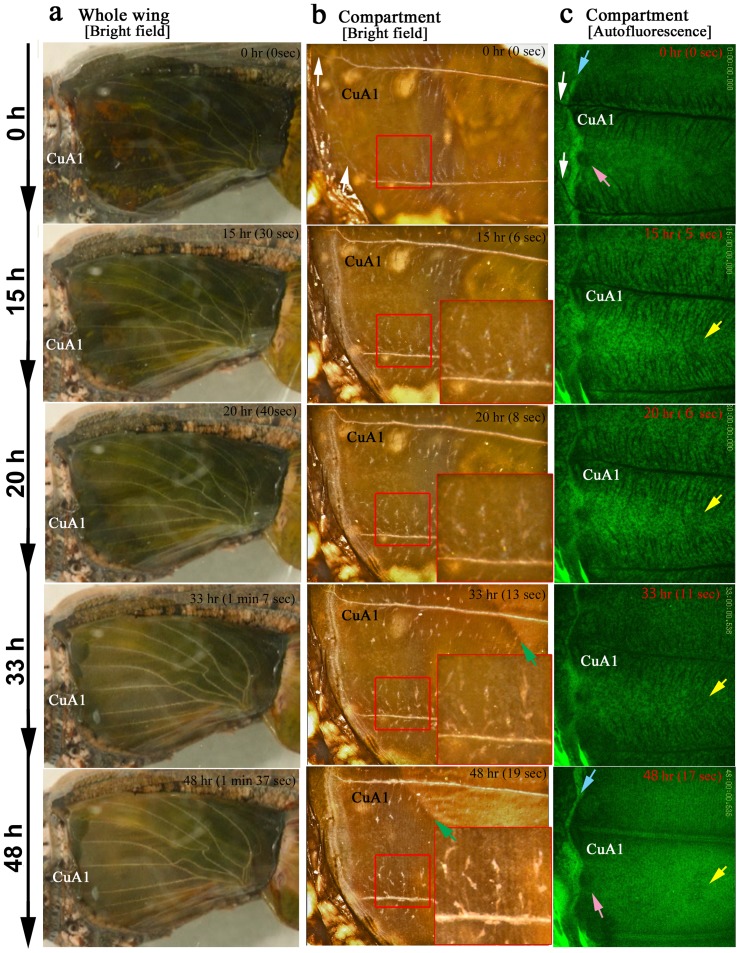
The changes in the pupal wing tissue in the first two days. The hours indicate real developmental time (i.e., the postpupation time), and the minutes and seconds in parentheses indicate time points in the playing movies. (**a**) Whole-wing bright-field images. The transparent tissue is becoming gray-white. Also refer to Movie S1. (**b**) Bright-field images of compartment CuA_1_. The small boxed areas are enlarged in the adjacent large boxes. The elaboration and movement of the tracheal branches are notable. Regular arrays of epithelial cells are observed by 48 h postpupation. The possible appression front moving from the basal to the distal region is indicated by green arrows. White arrows indicate the major tracheae that run off the wing edge at the early stage. Also refer to Movie S2. (**c**) Autofluorescent images of compartment CuA_1_ under blue light. Blue arrows indicate the bordering lacuna, which corresponds to the wing edge. White arrows indicate the major tracheae that run off the wing edge at the early stage. The pink arrows indicate the marginal focus (edge spot), which is the possible organizing center for the marginal band system. The yellow arrows indicate the location of the organizing center for a border symmetry system including an eyespot. The extension of tracheal branches, especially toward the organizing centers, is detectable. Moving hemocytes are observed in a relatively early stage, some of which are most likely macrophages. In the later stages, hemocytes are confined to the bordering lacuna, which may promote degradation of the peripheral tissue. Also refer to Movie S3.

At the compartmental level, we focused on the CuA_1_ (*n* = 5) and Sc+R_1_ (*n* = 4) compartments using a bright-field digital microscope (Figure 2a; Movie S2). The two compartments showed similar (if not identical) features. The elaboration and movement of numerous tracheal branches were notable, and numerous free-moving hemocytes [42] were observed underneath the epithelial sheet (Movies S2 and S4), as revealed in the autofluorescent images obtained using the confocal microscope as well (*n* = 3) (Movie S3). Regular arrays of epithelial cells were being generated when the entire hindwing became slightly gray-white at 20–33 h postpupation (Figure 2a–c). Array formation was completed by 48 h postpupation (Figure 2b, c). Then, the contraction pulses became more frequent, and the tracheal branches became less mobile (Movies S2 and S3). Together with the contraction pulses, a possible front of appression that may induce a seal between the dorsal and ventral epithelia moved from the basal to the distal region (Figure 2b), which likely accompanied the decrease of hemolymph space within the pupal wing [42]. It appeared that the compartment area became larger following the contraction pulses, which will be examined later in this paper.

In the autofluorescent images (*n* = 3), a possible organizing center for the marginal band system [3], [21] was observed immediately after pupation (0 h) in the form of a dark region at the prospective wing margin compared to other regions (Movie S3). This dark region corresponds to the marginal focus, which is specified by the edge spot of the pupal cuticle spots in a forewing [21]. A prospective eyespot focus, which corresponds to the organizing center for the border symmetry system, was similarly observed as a dark region almost immediately after pupation (0 h), and it appeared to be connected to more tracheal branches than other regions, which was observed beginning at 15 h postpupation (Figure 2c). After the movement of the tracheal branches gradually disappeared from the proximal to distal region, the prospective eyespot focus was observed more clearly as a dark region at 48 h postpupation (Figure 2c). At this point, the tracheal system could not be detected as non-fluorescent objects.

Interestingly, the major wing-vein-associated tracheae passed through the wing edge and the bordering lacuna at 0 h postpupation (Figure 2c). The extra portion was not eliminated (Movies S2 and S3). At the same time, the peripheral tissue appeared to begin degenerating, as demonstrated by previous studies [40]–[44]. However, in our images, the degeneration appeared to occur at the margin of the pupal wing proper at 35 h postpupation. Most likely due to the degeneration of the wing margin, the bordering lacuna expanded, and the marginal scales grew into the bordering lacuna. In the pupal wing proper, free-moving hemocytes were already observed at 0 h postpupation, then increased in number, peaking at 20–30 h postpupation. The hemocytes then gradually disappeared by 48 h postpupation, but they were still active in peripheral tissues that were degenerating (Figure 2c, Movie S3), which may simply be because hemolymph space was available only in the peripheral region at that time. This finding could suggest that some of the hematocytes may play a role in sequestering cell debris.

Regular arrangement of the epithelial cells occurred at approximately 32 h postpupation (Movies S2 and S3). The row-arrangement front moved from the proximal to the distal region. Following the arrangement, scale growth and expansion of the compartment area occurred. The growth of the marginal scales was notable. Furthermore, contraction pulses and folding of wing tissue took place at 75 h postpupation, which may be due to the growth of the tissue. The bordering lacuna disappeared due to the expansion of the pupal wing proper.

### Cellular Arrangement, Scale Growth, Tracheal Branches, and Hematocytes

**Figure 3:**
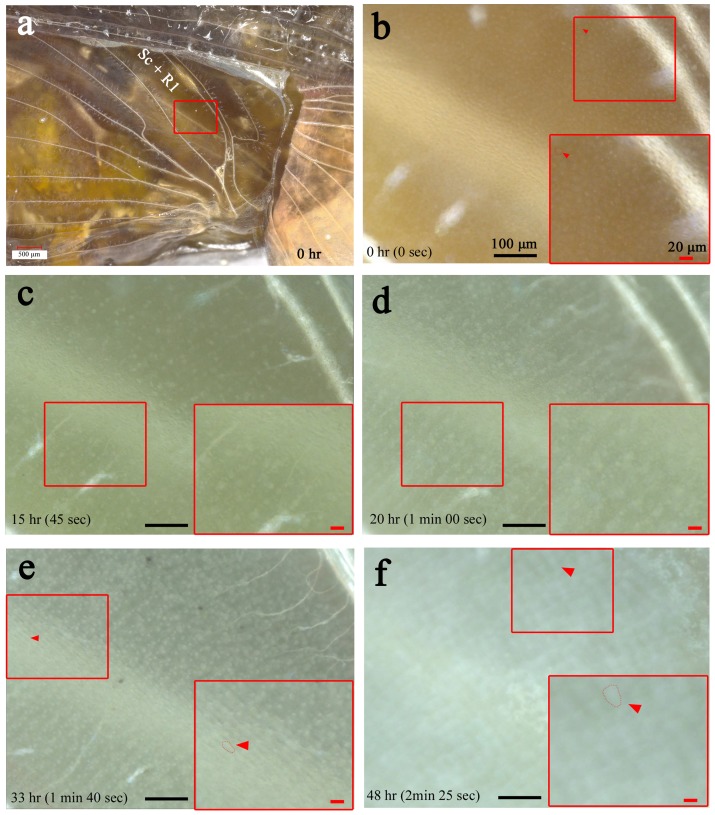
Array and scale formation. Also refer to Movie S4. (**a**) The hindwing basal region after the curling operation. The magnified area of compartment Sc+R_1_ in the subsequent panels is boxed. (**b-f**) Cellular changes over time. The small boxed areas are enlarged in the adjacent large boxes. Transparent non-aligned epithelial cells are observed in the early stage in (b). Vigorously moving tracheal branches are notable in (c), together with moving hemocytes, some of which are most likely macrophages. Later, the epithelial cells are regularly arranged, and scales grow, as shown in (d) and (e), which are seen as white objects that are increasing in size. Scale growth accompanies the increase in wing area in (f), which appears to be driven by the contraction pulses of the wing tissue. Red arrowheads indicate single cells or scales that are circled with dots. All panels (b-f) are shown at the same magnification.

**Figure 4:**
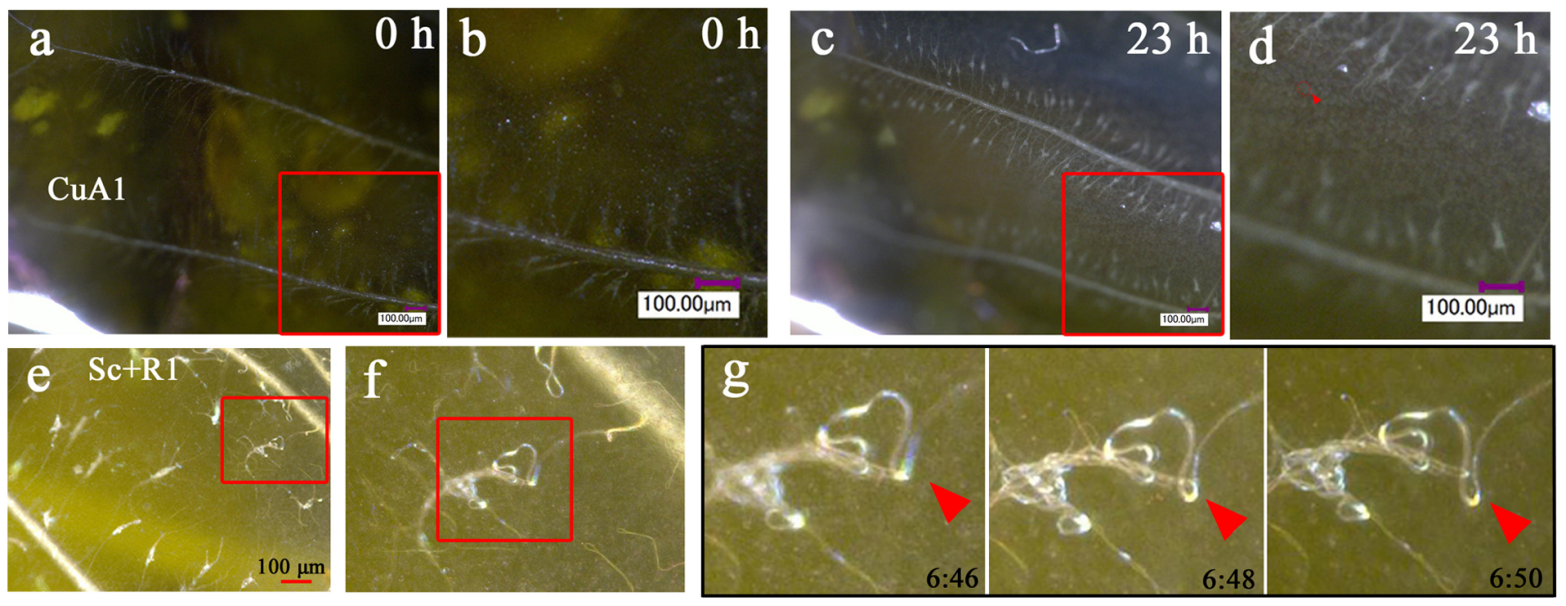
Behavior of tracheal branches and hemocytes. (**a, b**) Bright-field image of compartment CuA_1_ immediately after pupation (0 h postpupation). The boxed region is magnified in (b). Many tracheal branches are already observed at this point, but they are most likely immature and cannot be seen clearly under our observation conditions. They also do not move vigorously. (**c, d**) The identical CuA_1_ region 23 h postpupation. Many tracheal branches are observed as white crinoid-like objects, with one side attached to the major trachea. They move relatively vigorously. A single branch exhibits a white knob, from which many thin branches (i.e., tracheoles) radiate. Also note the free-moving hemocytes (indicated by a red arrowhead). (**e-g**) Tracheal branches in compartment Sc+R_1_. The boxed region in (e) is magnified in (f), and the boxed region in (f) is further magnified in (g), showing the dynamics of the branch, knob, and tracheoles. The red arrowheads in (g) indicate the identical position in the images over time. The postpupation time is indicated in (g).

Similar observations were made in the Sc+R_1_ and CuA_1_ compartments at higher magnifications using a bright-field digital microscope (*n* = 9) (Figures 3 and 4; Movie S4). A transparent cellular sheet of wing epithelium was observed immediately after pupation (Figure 3a, b). The cellular diameter was approximately 10 µm. At 15–20 h postpupation, the epithelial cells started to be regularly arranged (Figure 3c, d), and we successfully recorded growing scales at approximately 33 h postpupation afterwards (Figure 3e). The scales subsequently increased in size, while the wing area increased dramatically (Figure 3f).

Free-moving hemocytes were clearly observed underneath the epithelial sheet at approximately 1 d (23 h) after pupation (Figure 4). Tracheal branches were also present immediately after pupation, but they were not yet well elaborated (Figures 3 and 4). The tracheal branches were numerous and moved briskly (Figures 3 and 4). A single branch from the major trachea exhibited a crinoid-like structure, consisting of a stalk with a knob from which tracheoles radiate. These knobs and tracheoles appeared to be attached to the epithelial sheet from inside, but their columnar stalk did not. The knobs became less obvious as the epithelial cells were arranged.

### Cellular Fluorescent Staining of Wing Tissues

**Figure 5:**
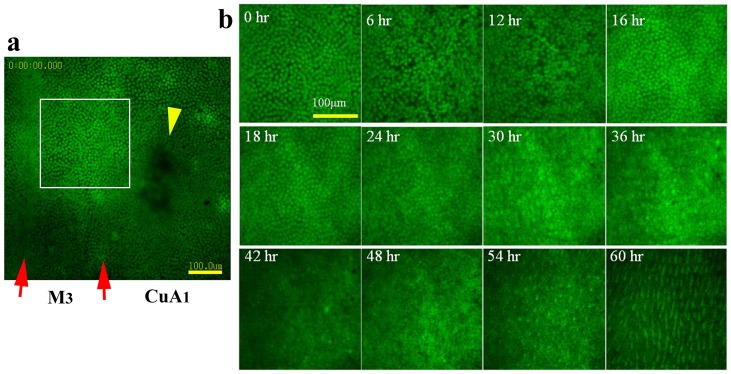
Fluorescent images of array and scale formation. Wing tissue was stained with CFSE. (**a**) Stained M_3_ and CuA_1_ compartments. Compartment CuA_1_ exhibits an organizing center, indicated by a yellow arrowhead. The organizing center appears to be resistant to staining. Note that the adjacent M_3_ compartment does not have this non-stained black area, probably because the M_3_ compartment does not have an eyespot in adult wings. Also see Figure 6a. Red arrows indicate the major tracheae. The boxed region is enlarged in the subsequent panels. Also refer to Movie S5. (**b**) Cellular changes over time. At 6 h, the cellular density appears to decrease, and by 16 h the tissue is occupied by densely packed epithelial cells. After 24 h, the cells gradually become arranged, and the wing area increases, which appears to be driven by the contraction pulses that become frequent after 30 h. Scale growth is observed immediately after the cellular row arrangement occurs. All panels are shown at the same magnification.

To confirm the dynamics of cellular arrangement using fluorescent images, we performed CFSE staining of wing tissues (*n* = 2) (Figure 5; Movie S5). The organizing center for the border symmetry system (i.e., eyespots and their associated elements) in the CuA_1_ compartment did not stain well. The adjacent compartment (compartment M_3_), which does not contain an eyespot, was relatively evenly stained, and we therefore focused on this compartment. Immediately after pupation, epithelial cells were observed, which were not yet arranged and were loosely distributed. At approximately 6 h postpupation, black patches appeared randomly, which may indicate cellular gaps due to cellular mobility or division. By 16 h postpupation, the black patches had disappeared, and cells were packed relatively tightly. These cells appeared to be smaller than those at 0–6 h, suggesting that cell division occurred. Gradually, cellular arrangement occurred, beginning at approximately 24 h postpupation, after which scales appeared to develop. By approximately 54 h postpupation, many fluorescent dots emerged, which indicated growing scales. Careful examination of the movie indicated that the arrangement and scale development occurred from the proximal to distal region (from the top to the bottom in Figure 5 and Movie S5).

**Figure 6:**
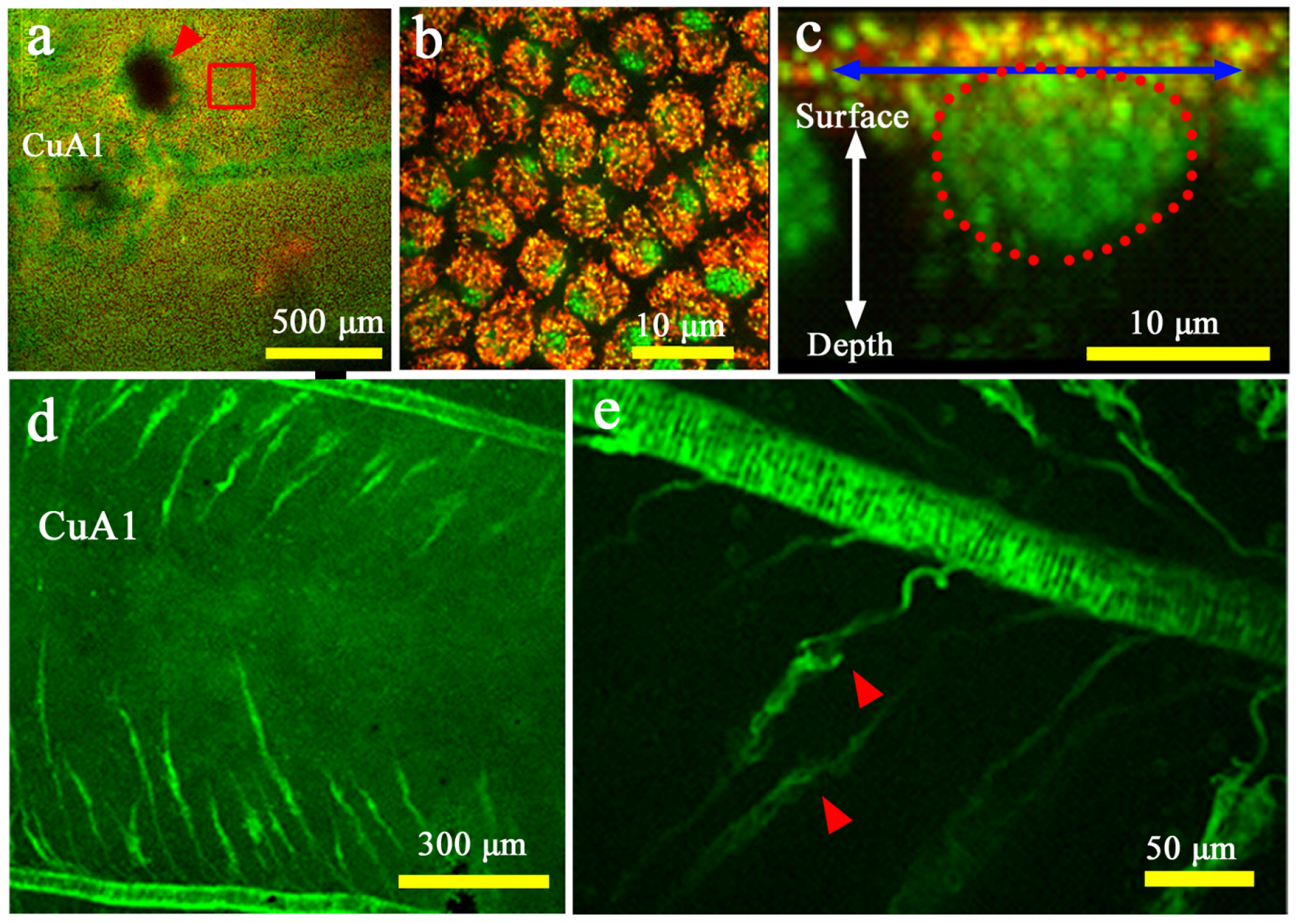
Fluorescent images of epithelial cells and tracheal branches. (**a**) Low-magnification image of compartments CuA_1_ and M_3_ stained with MitoTracker Orange for mitochondria (in red) and SYBR Green-1 for nuclei (in green), immediately after pupation. The red arrowhead indicates the organizing center for the border symmetry system, which is resistant to staining. This non-stained black area was not detected in the adjacent compartment where no eyespot exists in adult wings. Also see Figure 5a. The boxed region is magnified in (b). (**b**) High-magnification image of the epithelial cells boxed in (a). Yellow dots indicate mitochondrial DNA. (**c**) Optical cross section of the stained epithelial cells. The diameter of a single cell is indicated by a double-headed arrow. Cellular nucleus is encircled by red dots. Note the mitochondrial distribution on the dorsal surface of the cell. Yellow regions indicate mitochondrial DNA. (**d, e**) Compartment CuA_1_ stained with DiBAC_4_(3). Tracheae are stained well, and the epithelial cells are weakly stained. A portion of a tracheal knob is indicated by red arrowheads to demonstrate its vigorous movement.

To confirm the identity of the epithelial cells and their relationship with the tracheal branches, we performed double staining of living tissues using two fluorescent dyes, MitoTracker Orange for mitochondria and SYBR Green-1 for nuclei (*n* = 7). We observed developing cells in the compartment CuA_1_ at 0.5 h postpupation (Figure 6a–c). The cellular diameter was approximately 10 µm, which was consistent with the above results obtained using the bright-field digital microscope. Interestingly, the organizing center was resistant to staining. Stacking of the *Z*-axis images revealed that the dorsal surface of the epithelial cells was rich in mitochondria above a relatively large nucleus (Figure 6c). We also found that DiBAC_4_(3) strongly stained the major wing-vein-associated tracheae and tracheal branches and weakly stained epithelial cells (*n* = 4) (Figure 6d, e).

### Contraction Pulses of the Developing Hindwing

Low-frequency slow contraction pulses were observed prior to the epithelial cellular differentiation indicated by the gray-white coloration of the wing tissue. Although the mechanism underlying these contraction pulses is unknown, it appeared that they were either tissue autonomous or driven by thoracic flight muscle. We found that both the fore- and hindwings showed synchronized pulses (Movie S1).

**Figure 7:**
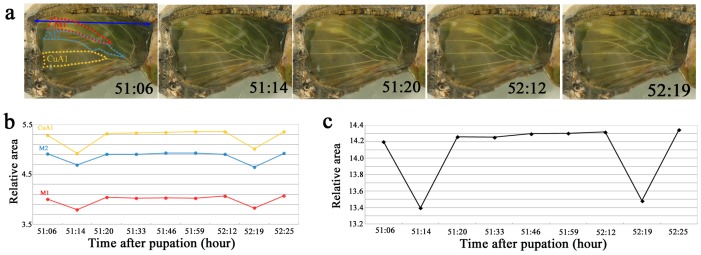
Areal changes associated with the wing-tissue contraction pulses. (**a**) Bright-field images of the hindwing over time. The postpupation time is indicated in each panel. The areas of the three different compartments (M_1_, M_2_, and CuA_1_), encircled with red, blue, and yellow dotted lines, were measured and normalized according to the hindwing span indicated by the double-headed blue arrow. Note that the hindwing is most contracted at 51∶14 h and at 52∶19 h postpupation. These are static images from Movie S1. (**b**) Relative areal changes over time in compartments M_1_, M_2_, and CuA_1_. The time points correspond to the images shown in (a). These measurements were performed using Movie S1. (**c**) Summation of the three compartment areas.

Because the contraction pulses appeared to be associated with the wing-tissue expansion, we quantitatively examined whether the wing area values changed before and after the contraction pulses using the bright-field whole-wing images. During a contraction, the areas of compartments M_1_, M_2_, and CuA_1_ decreased and then increased back to similar or slightly greater areas (Figure 7). However, because there were slight increases in area observed without contraction, we do not know whether the size increase following a contraction was directly due to the contraction.

**Figure 8:**
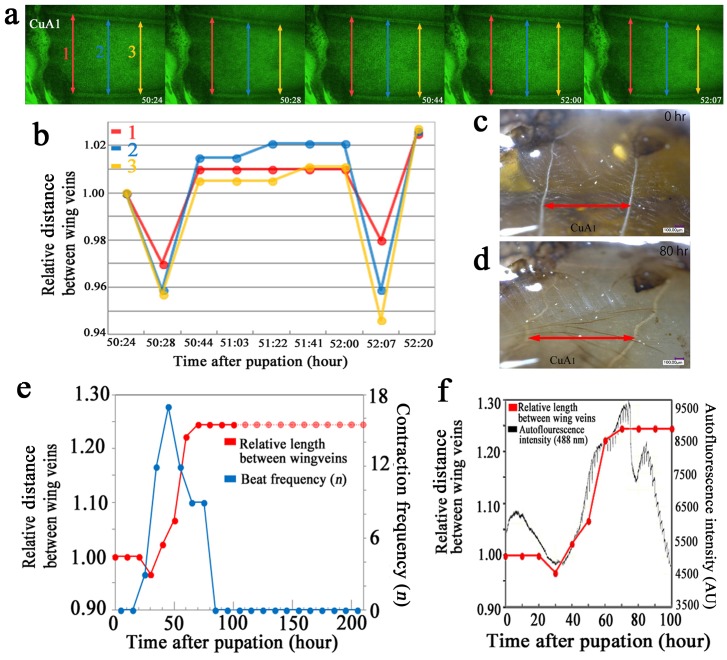
Distance changes associated with the wing-tissue contraction pulses and autofluorescence intensity. (**a**) Three distance measurements (red, blue, and yellow double-headed arrows, designated 1, 2, and 3, respectively) between the two veins that define compartment CuA_1_. The postpupation time is also indicated. All panels are shown at the same magnification. These are static images from Movie S3. (**b**) Changes in length (distance) between the two wing veins over time. The measured distances are indicated in (a). These measurements were conducted using Movie S3. (**c, d**) Images of the distances between the wing veins at 0 h and 80 h postpupation in compartment CuA_1_. These panels are at the same magnification, showing static images from Movie S6. These images of the wing of a physically fixed pupa were taken at the same position at different time points, and the distances were measured between the two points despite the tilting of the wing veins relative to the double-headed red arrows as the wing grows. (**e**) Relative distance between the wing veins in compartment CuA_1_, as shown in (c, d) (indicated with red dots and lines), and the contraction frequency (shown in blue dots and lines). The distance at 0 h was considered to be 1.00, and other values were normalized accordingly. The broken lines indicate difficulty in measuring the distance, but no change appeared to occur afterwards. Note that an increase of the contraction frequency is followed by an increase in this distance in the early phase, and a decrease of the beat frequency is followed by the distance plateauing. These measurements were conducted using images from Movie S6. (**f**) The relative distance between the wing veins, as shown in (e) (measured from Movie S6), and the autofluorescence intensity (measured from Movie S3). The autofluorescence intensity increases together with the relative length between the wing veins.

Similar results were obtained using autofluorescent images of developing wings. We measured the distances between two wing veins and found that the distance decreased during a contraction and then increased back to the original or a slightly greater distance (Figure 8a, b). When the distances were compared between 0 and 80 h postpupation, the latter distance was greater (Figure 8c, d).

To examine the relationship between the contraction pulses and the increase in vein distances, we recorded the frequency of contractions (the number of contractions within a 10-h period) and the wing-vein distances every 10 h. The contraction frequency was generally low, but it was highest (17 times) in the 40–50 h period, which coincided with the period of a marked increase in vein distance (Figure 8e). This period also coincided with the period of increasing scale size. Furthermore, the period during which the vein distance increase occurred coincided with the period of increased autofluorescence (Figure 8f).

### Color Pigment Deposition in Scales

**Figure 9:**
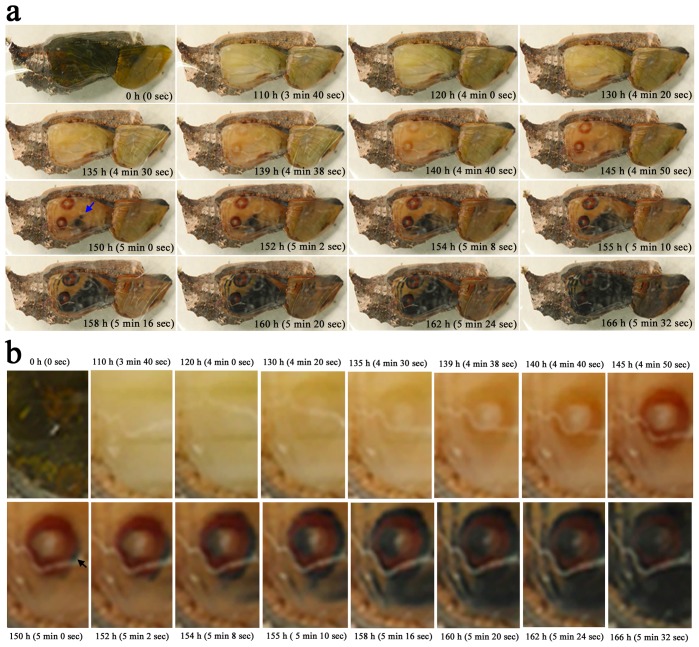
Ontogeny of the pigment deposition process at the whole-wing level. Real time points are shown along with the playing time points from Movie S1 (in parentheses). (**a**) Changes in the whole wing. The background black coloration emerges at the center of the wing (indicated by an arrow at 150 h). (**b**) High-magnification images of the eyespot. The red ring is produced first, and the black ring is produced at a single location at 150 h (indicated by an arrow). The black ring then develops around the red ring. Later, the width of the black ring expands considerably.

We analyzed the time course of pigment deposition in a single wing of a given individual in whole-wing images (*n* = 3) (Figure 9; Movie S1). Color patterns (including eyespots and parafocal and submarginal bands) were already recognizable approximately 2 d before eclosion (approximately 5 d after pupation), but pigments were clearly deposited approximately 1.5 d before eclosion. This pigment deposition process continued until the point immediately prior to eclosion.

Coloration appeared in the following order: eyespot red rings, black/brown background (from the discal spot or its proximity to the peripheral area), eyespot black rings, and black peripheral elements (the parafocal and submarginal bands). However, the black rings and the black peripheral elements were detected as early as the red rings. The background black/brown coloration extended from a discal spot or its proximity toward the distal and proximal regions, and this expansion appeared to be blocked by the eyespots and the parafocal elements. The black ring first emerged at a single location and expanded along the red ring. Later, the width of the black ring expanded considerably. This sequence of events was not observed clearly in the red ring.

**Figure 10:**
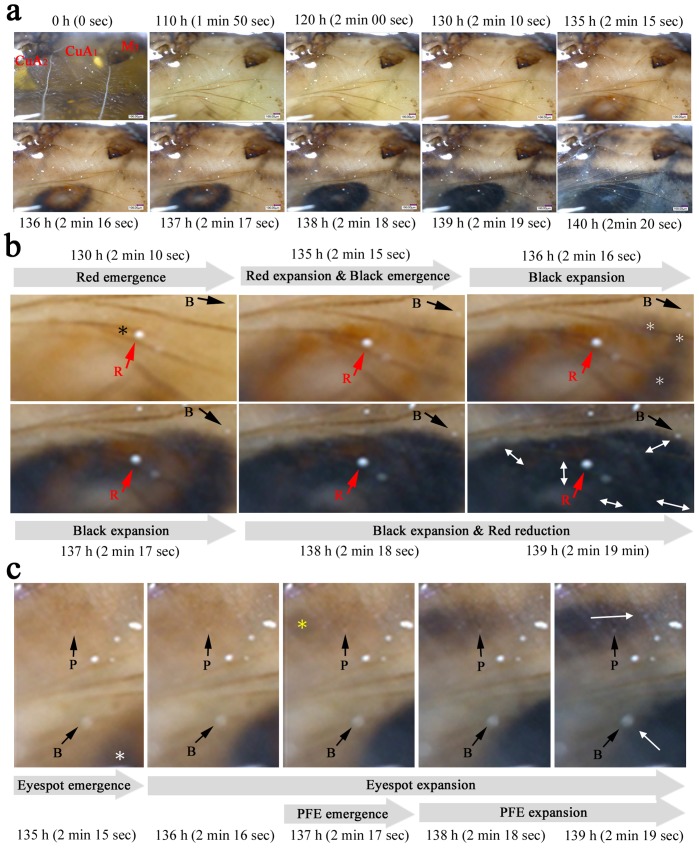
Ontogeny of the pigment deposition process at the compartmental level. Real time points are shown along with the playing time points from Movie S6 (in parentheses). (**a**) The changes in CuA_1_ and its adjacent compartments. (**b**) High-magnification images of CuA_1_ compartment. The eyespot red ring develops first. A possible red fragment is indicated by a black asterisk at 130 h. Black fragments are indicated by white asterisks at 136 h. Black and red arrows indicate artifactual objects that can be used as physical reference points. Reference point R is located in the middle of the red ring at 135 h but is gradually invaded by the black ring. Reference point B is located far from the black ring at 136 h, but the black ring reaches it at 139 h. The directions of black ring expansion are indicated by double-headed white arrows in the image from 139 h. (**c**) Another high-magnification image. Reference point B is located far from the black fragment (indicated by a white asterisk) at 135 h, but the black region reaches it at 139 h. Reference point P is located far from the black region at the center of the prospective parafocal element (indicated by a yellow asterisk), but the black region later expands laterally to cover the reference point P entirely. The directions of expansion of the black pigment are indicated by white arrows in the image from 139 h.

We further analyzed the pigment deposition sequence using the compartmental images (*n* = 5) (Figure 10; Movie S6), which revealed that within a prospective width of the black ring, the black pigment was first deposited in patches as fragments in the central areas of the width. The pigmented regions of the black ring then expanded not only along the red ring but also outside and inside the eyespot (Figure 10a, b). Similarly, within a prospective parafocal element, the black pigment was first deposited at the center, and it expanded laterally (Figure 10a, c). The red ring that appeared first was invaded by the black ring that developed subsequently. This superimposition process reduced the transient width of the red ring considerably.

## Discussion

In this study, we obtained real-time *in vivo* images of butterfly wing tissue development. The developmental time-course that was observed in this study is largely consistent with and complementary to those found in previous histological and morphometric studies on lepidopteran wings [1], [4]–[8], [35]–[47]. We recorded time course of developing epithelial cells, including the arrangement of rows and scale growth. We also described images of epithelial cells that are rich in mitochondria at the dorsal surface of the cell. Mitochondria may be functionally important in these actively growing and differentiating epithelial cells to supply ATP as a free energy source. The mitochondrial distribution pattern at the surface of epithelial cells may suggest a high metabolic activity for producing scales at the surface of the epithelium.

Consistent with the observed richness of mitochondria, we found briskly moving tracheal branches originating from the major tracheae, which supply oxygen to epithelial cells from inside the tissue. The structure of a single tracheal branch is crinoid-like, similar to a sea lily, exhibiting a white knob from which many tracheoles radiate. Interestingly, the prospective eyespot focus is likely to be connected to more tracheal braches than other regions, and its autofluorescent character and sensitivity to fluorescent dyes are different from other regions, as observed in the putative marginal focus (edge-spot) organizing centers. The optical character of the organizing centers may be due to high density of cells that formed a non-flat structure in wings (i.e., an elevation or dent of wing surface), which is consistent with the previous report [47]. These unique features of the organizing centers may indicate their high metabolic activity related to signaling.

In addition to the highly mobile tracheal branches, we discovered cellular dynamics that may be difficult to detect using fixed histological samples, including free-moving hemocytes; slow, low-frequency contraction pulses; movement of the possible appression front in the early pupal stage; and dynamic coloration processes a few days prior to eclosion. Within one day after pupation, moving hemocytes were observed underneath the epithelial sheet. Initially, the hemocytes were seen throughout a compartment and were not confined to the lacunae along tracheae, indicating the existence of a hemolymph space between the two epithelia at this point. Later, the hemocytes were confined to the bordering lacuna. Some of these hemocytes may be macrophages and may be responsible for eliminating apoptotic cells from the wing tissue and for degradation of the peripheral region outside the pupal wing proper. Interestingly, we observed similar macrophage-like cells during the damage-induced regeneration of fish skin [48]. In our images, degradation of the margin of the pupal wing proper widened the bordering lacuna.

We discovered contraction pulses in the wing tissue. A single pulse cycle required 10–20 min, and the highest frequency recorded was 17 pulses within a 10-hour period, meaning that one contraction occurred every 35 min on average. Because such slow, low-frequency contractions are difficult to detect in fixed histological samples, the present study is likely the first report of these contraction pulses. These contractions are too slow and too rare in frequency to be caused by the heart or accessory pulsatile organs. Thoracic flight muscles may be involved in the contraction process. However, because the contraction is very slow and because it is coupled with the wing area increase and with the scale development, there is a possibility that the wing contraction may be autonomous. Although the functional importance of the contraction pulses is unclear, they appeared to be coupled to increases in the wing area and autofluorescence intensity. This wing-area increase may have been achieved through increases in cell size and by the growth of scales on the surface of the wing, as cell division likely ceased prior to the arrangement of rows [36], [37]. Further physiological characterization of these contractions can be expected in the future.

Butterfly wings are a two-dimensional system on which scales (and hence scale and socket cells) are regularly arranged in anteroposterior rows at regular intervals [1], [4]–[8], [45], [46]. In the present study, we found that the arrangement of the cellular rows began at approximately 20 h postpupation. Almost simultaneously, scales began to grow. Initially, the scales were colorless and were observed as white or fluorescing objects. At this point, it is likely that positional information has already been supplied from organizing centers and that the scale-forming cells have committed to their fate regarding scale coloration, size, and shape [45], [46]. Scale size likely reflects the degree of polyploidy in the scale cells of butterflies [1], [49]–[51]. Cell size and shape may be determined by positional information, which may be supplied as a ploidy signal [46]. Unfortunately, the present study did not have a sufficient resolving power to detect changes in cell size.

Nevertheless, we present a detailed time course of color pigment deposition in the scales of a single individual. As indicated in a previous study [45], the background coloration of this species shows similar behavior to an enlarged element, as it originates from the discal spot or its proximity, which is the center of the central symmetry system. Furthermore, the peripheral region is not invaded by this black/brown coloration, most likely because the pigmentation front is blocked by the eyespots and parafocal elements. These phenomena are reminiscent of elemental interactions between an eyespot and a parafocal element [22] and between two eyespots [19].

An alternative explanation is that the pigment deposition order and pigment intensity simply reflect either the distribution pattern of pigment synthesizing enzymes or the availability of pigment precursors provided via hemolymph. However, it appears that the black pigment was first deposited at the place where the primary organizing center is located, namely, the discal spot. Furthermore, the distribution pattern of pigment synthesizing enzymes is likely what positional information for color patterns determines. The availability of pigment precursors would be basically uniform throughout a wing, and it is difficult to think that the prospective discal spot organizing center is more accessible to pigment precursors than other portions of a wing.

In the whole-wing images, the black ring appeared at the single location first and then expanded along the red ring. The black ring further expanded in width. In the case of the red ring, such a sequence of events was less apparent. The expansion of the black ring was further observed in the compartmental images, which revealed the fragmental or patchy deposition and expansion. That is, the black fragments emerged first and then expanded laterally, distally and proximally to complete a ring. The outer parts of the red ring were subsequently invaded by the expanding black ring, indicating that the red signal and the black signal overlap in the boundary area. In other words, the two signals in a given eyespot are different from each other. The development of the parafocal element followed a similar sequence, although only a single fragment emerged first at the center of the parafocal element and expanded laterally.

We believe that these detailed analyses of the pigment deposition process are insightful for understanding the nature of positional information. The process of determination of positional information (i.e., pre-patterning) occurs immediately after pupation, and the process of pigment deposition occurs immediately before pupation. Therefore, these two processes are different. However, the latter must be executed according to the former. In this sense, these two processes are closely related. We believe that increased enzymatic activity related to pigment synthesis may be indicated by the timing of the appearance of coloration during development, and we can further assume that this difference in activity reflects the difference in positional information, i.e., different levels of morphogenic signals.

We have proposed the induction model as a mechanism for specifying positional information [19], [20], [24], [52], [53], which is more widely applicable to various color patterns than the classical morphogen gradient model. The induction model states that a pulse-like signal for a dark eyespot ring (a dark signal) is emitted from the prospective focus. The velocity of the signal decreases as it goes further. Cells that are located in positions where the dark signal settles can serve as a secondary organizing center by amplifying the signal itself. This nested dark signal also induces a different signal for a light eyespot ring (a light signal) adjacent to it. This light signal is inhibitory to the dark signal. Dynamic interactions among these dark and light signals determine the final eyespot morphology. We were able to observe the time course of pigment deposition in a wing from a given individual, and if the pigment deposition process reflects the distribution pattern of positional information, our observations support the induction model of positional information, which predicts central-to-peripheral deposition (due to independent nested signaling from the center of a width of a black ring or from the center of a parafocal element) that can be executed in a patchy manner. Our observations are not consistent with the gradient model of positional information because it predicts even deposition (due to an even threshold-like response within a width of a black ring or within a parafocal element). It is plausible that a parafocal element is equivalent to an isolated black ring of an eyespot, as predicted by the previous analyses [3], [20], [22], [24], [33], [45], [46]. Furthermore, the superimposition of a part of a black ring on a red ring cannot be supported by the gradient model, because redundant information for a single position cannot theoretically be supplied by the gradient model. However, if the pigment deposition process does not reflect the distribution pattern of positional information at all, these observations support neither the induction model nor the gradient model. It is interesting to note that a recent study showed that morphogen molecules do not have to form concentration gradients to function [54].

Technically, real-time *in vivo* imaging is an entirely new approach for investigating butterfly biology. In the present study, we employed several techniques, including three different optical systems, and took advantage of inherent characters of butterflies, such as their resistance to surgical operation, the autofluorescence of developing cells, and the penetration of fluorescent dyes. The time points detected using the different systems are slightly different but largely consistent. Each system exhibits advantages and disadvantages. Although we utilized only a handful of fluorescent dyes in this study, we believe that the application of other dyes may increase our observational range. Genetically engineered fluorescent proteins may also be employed, as a gene transfer method is now established [55]. In the future, we may be able to trace molecular movements in real time in a developing wing using this system, which may provide us with conclusive evidence of how morphogens determine eyespots and other color pattern elements in butterfly wings.

## Supporting Information

Movie S1 **Whole-wing bright-field imaging of a** ***J. orithya*** **pupa.**
(MP4)

Movie S2 **Compartmental bright-field imaging of pupal wing tissue.**
(MP4)

Movie S3 **Compartmental autofluorescent imaging of pupal wing tissue.**
(MP4)

Movie S4 **High-magnification compartmental bright-field imaging of pupal wing tissue.**
(MP4)

Movie S5 **CFSE fluorescent imaging of pupal wing tissue.**
(MP4)

Movie S6 **Time course of the color pigment deposition process in pupal wing tissue.**
(MP4)
